# Trends in absolute and relative educational inequalities in four modifiable ischaemic heart disease risk factors: repeated cross-sectional surveys from the Nord-Trøndelag Health Study (HUNT) 1984–2008

**DOI:** 10.1186/1471-2458-12-266

**Published:** 2012-04-03

**Authors:** Linda Ernstsen, Bjørn Heine Strand, Sara Marie Nilsen, Geir Arild Espnes, Steinar Krokstad

**Affiliations:** 1 Faculty of Nursing, Sør-Trøndelag University College, Mauritz Hansens gt 2, 7004, Trondheim, Norway; 2 Research Centre for Health Promotion and Resources HIST/NTNU, Trondheim, Norway; 3 The Liaison Committee between the Central Norway Regional Health Authority and The Norwegian University of Science and Technology, Trondheim, Norway; 4 Norwegian Institute of Public Health, Marcus Thranes gt 6, 0473, Oslo, Norway; 5 Department of Public Health and General Practice, Faculty of Medicine, Norwegian University of Science and Technology, Trondheim, Norway; 6 Department of Social Work and Health Science, Norwegian University of Science and Technology, Trondheim, Norway; 7 HUNT Research Centre, Department of Public Health and General Practice, Faculty of Medicine, Norwegian University of Science and Technology, Levanger, Norway; 8 Levanger Hospital, Health Trust Nord-Trøndelag, Levanger, Norway

**Keywords:** Trends, Socioeconomic inequalities, Gender differences, Ischaemic heart disease risk factors, Smoking, Diabetes, Hypertension, High total cholesterol

## Abstract

**Background:**

There has been an overall decrease in incident ischaemic heart disease (IHD), but the reduction in IHD risk factors has been greater among those with higher social position. Increased social inequalities in IHD mortality in Scandinavian countries is often referred to as the Scandinavian “public health puzzle”. The objective of this study was to examine trends in absolute and relative educational inequalities in four modifiable ischaemic heart disease risk factors (smoking, diabetes, hypertension and high total cholesterol) over the last three decades among Norwegian middle-aged women and men.

**Methods:**

Population-based, cross-sectional data from The Nord-Trøndelag Health Study (HUNT): HUNT 1 (1984–1986), HUNT 2 (1995–1997) and HUNT 3 (2006–2008), women and men 40–59 years old. Educational inequalities were assessed using the Slope Index of Inequality (SII) and The Relative Index of Inequality (RII).

**Results:**

Smoking prevalence increased for all education groups among women and decreased in men. Relative and absolute educational inequalities in smoking widened in both genders, with significantly higher absolute inequalities among women than men in the two last surveys. Diabetes prevalence increased in all groups. Relative inequalities in diabetes were stable, while absolute inequalities increased both among women (p = 0.05) and among men (p = 0.01). Hypertension prevalence decreased in all groups. Relative inequalities in hypertension widened over time in both genders. However, absolute inequalities in hypertension decreased among women (p = 0.05) and were stable among men (p = 0.33). For high total cholesterol relative and absolute inequalities remained stable in both genders.

**Conclusion:**

Widening absolute educational inequalities in smoking and diabetes over the last three decades gives rise to concern. The mechanisms behind these results are less clear, and future studies are needed to assess if educational inequalities in secondary prevention of IHD are larger compared to educational inequalities in primary prevention of IHD. Continued monitoring of IHD risk factors at the population level is therefore warranted. The results emphasise the need for public health efforts to prevent future burdens of life-style-related diseases and to avoid further widening in socioeconomic inequalities in IHD mortality in Norway, especially among women.

## Background

Of the 17.1 million global deaths from cardiovascular diseases in 2004, ischaemic heart disease (IHD) constituted the largest group with 7.2 million (42%) deaths [1]. Despite overall decline in IHD mortality in developed countries during the last decades, increased social inequalities in IHD mortality are observed both between and within the Nordic countries, particularly in women [2,3]. In the Nordic countries, decreasing IHD mortality rates are explained as an effect of both reduced disease incidence and better treatment [4,5]. Primary prevention with reduced risk factor levels was found to explain half of the decline in IHD mortality in the USA [6], which indicates that risk factor levels play an important role in trends of IHD incidence.

In the large INTERHEART case–control study, Yusuf et al. [7] found that smoking, cholesterol, hypertension and diabetes predicted 76% of the population attributable risk of myocardial infarction and that this proportion varied little across continents. Public health initiatives over the last decades might have resulted in reduced prevalence in some of these modifiable risk factors. However, cultural factors and social changes could drive levels and distributions of some IHD risk factors in undesirable directions. Diabetes is increasing worldwide primarily because of the development of a sedentary lifestyle with less physical activity and increasing obesity. Diabetes also appears to be a strong predictor for IHD development [7], especially in women [8,9], and a continued social gradient in the prevalence of diabetes [10] will most likely contribute to future trends in social inequalities in IHD mortality. Further, a sustained high intake of saturated fat and trans fat, which are typically found in fast food, will affect cholesterol levels. Recent studies confirm a social gradient in dietary habits [11] with higher total fat intakes among socioeconomically disadvantaged groups [12]. Smoking is one of the main causes of IHD worldwide [7] and smoking, as diabetes, seems to confer a stronger risk factor in women compared to men [13].

The more unfavourable trends in risk factors among women is a suggested mechanism behind the recent finding of increasing trends in incidences of myocardial infarction among Norwegian middle-aged women [14]. Educational level, as a measure of socioeconomic position, is strongly associated with IHD mortality in Norway [15] as in the rest of Europe [3]. Thus, widening or narrowing trends in socioeconomic inequalities in IHD development in women and men depend on trends in different risk factor levels, in particular the prevalence of smoking among the least educated [16,17].

Increased social inequalities in mortality in Norway [18], a country with a social democratic welfare regime are referred to as a “public health puzzle”; however, the magnitude of inequalities depends on measurement used [19]. Most notably, increasing relative but decreasing absolute inequalities is observed when the rate of improvement is smaller for those in the lowest social position. Most trend studies have focused on relative rather than absolute inequalities [19]. There is an ongoing discussion on what measure to use [20], but the general consensus is that both absolute and relative measures are needed to describe social inequalities [20,21]. It follows that monitoring both absolute and relative educational inequalities in classical IHD risk factors is important for understanding trends in the social distribution of IHD and for necessary public health initiatives to be taken.

The aim of this study was to examine secular absolute and relative trends in educational inequalities of four major modifiable IHD risk factors (smoking, diabetes, hypertension and high total cholesterol) in Norwegian middle aged women and men over three decades.

## Methods

### Study population

The Nord-Trøndelag Health Study (HUNT) is a Norwegian population based general health survey [22,23] conducted in 1984–86 (HUNT 1), 1995–97 (HUNT 2) and 2006–08 (HUNT 3). All persons aged 20 years and older (85,100 in HUNT 1; 94,194 in HUNT 2; and 93,210 in HUNT 3) were invited to participate. The overall participation rates decreased from 88% in HUNT 1 to 54% in HUNT 3. The participation was highest among the middle aged. To maintain comparability across all three surveys and to maximise numbers of participants, especially in HUNT 3, we limited our analyses to respondents aged 40–59 years with complete data on educational level and the four IHD risk factors. Response rates among those aged 40–59 years ranged from 93% in HUNT 1 to 79% in HUNT 2 and 60% in HUNT 3. Data were collected from questionnaires, blood samples and clinical measurements [23]. Blood samples were not collected in HUNT 1 (1984–1986). Because of missing data on education and the IHD risk factors under study we excluded 21.0% (n = 4,120) from HUNT 1, 5.7% (n = 1,355) from HUNT 2 and 16.0% (n = 3,420) from HUNT 3. The final samples included in the analyses consisted of n = 19,263 (HUNT 1), n = 23,658 (HUNT 2) and n = 17,973 (HUNT 3).

### Education as a proxy of social position

Education was selected as our indicator of socioeconomic position, given that it is attained relatively early in life and it is stable over the adult life span, and that education forms the steepest social gradient in IHD mortality in Norway [15]. Additionally, it is applicable for those not in the active labour force [24]. In the INTERHEART case–control study, education as a proxy for socioeconomic position was found to be most consistently associated with increased risk for acute myocardial infarction globally, especially in high-income countries [25]. Further, most studies confirm a significant link between education and health behaviour, and the association between education and IHD mortality is stronger than occupational position or income-based measures [26].

Data on educational level was retrieved from Statistics Norway. Using the International Standard Classification of Education (ISECD-97) [27] we collapsed the seven levels of education to three main levels: primary (primary and lower secondary school), secondary (upper secondary and post secondary school) and tertiary (first and second stage of tertiary education).

### IHD risk factors

*Hypertension* was defined as systolic blood pressure ≥140 mmHg or diastolic blood pressure ≥90 mmHg or as self-report of current use of antihypertensive medication. At HUNT 1 resting blood pressure was measured twice using a sphygmomanometer; the second measurement was used in this study. At HUNT 2 and HUNT 3 resting blood pressure was measured three times by a Dinamap 845 XT (Critikon) based on oscillometry. Blood pressure based on the mean of the second and third measurement was used in this study. In comparison with blood pressure measurement by a sphygmomanometer (HUNT 1), the use of Dinamap shows approximately the same levels for systolic pressure but slightly lower levels for diastolic pressure. *High total serum cholesterol* was defined as total serum cholesterol ≥ 5.0 mmol/L [28]. There were no questions about use of lipid-lowering drugs, thus medical treatment was not taken into account in the categorisation of hyperlipidaemia. Total serum cholesterol was analysed at the Central Laboratory at Levanger Hospital, using a Hitachi 911 Autoanalyzer (Hitachi, Mito, Japan) applying reagents from Boehringer Mannheim (Mannheim, Germany) [23]. *Smokers* were defined as those who consumed cigarettes, pipes or cigars on a daily basis. *Diabetes* was determined by a positive response to the question “Do you have or have you had diabetes”?

### Statistical methods

Age standardized prevalences of the IHD risk factors was calculated using 5 year age groups, the standard population being women and men 40–59 years old as of the 1^st^ of January 1999 in the Nord-Trøndelag county. All analyses were stratified by gender.

To measure the magnitude of relative and absolute educational inequalities in the four IHD risk factors we calculated the Relative Index of Inequality (RII) and Slope Index of Inequality (SII). RII and SII are summary measures recommended when making comparisons over time or across populations [29]. These indices are regression based and take the whole socioeconomic distribution into account, rather than only comparing the two most extreme groups. Educational level at each survey is transformed into a summary measure that is scaled from zero (highest level of education) to one (lowest level of education) and is weighted to reflect the share of the sample at each educational level. The population in each education category is assigned a modified ridit-score based on the midpoint of the range in the cumulative distribution of the population of participants in the given category. For example, if the most educated women comprise 18% of the population, the range of women in this category is assigned a value of 0.09 (0.18/2), and if the second category comprises 50% of the population, the corresponding value is 0.43 (0.18 + [0.5/2]) and so forth.

As suggested in the literature [30-32], we used generalised linear models (log-binomial regression), with a logarithmic link function to calculate RIIs (rate ratios) and with an identity link function to calculate SIIs (rate differences) [32]. Both indices were estimated with 95% confidence intervals with the following generalised linear model:

(1) $$g\left(Y\right)=constant+\beta_{1}ridit+\beta_{2}survey+\beta_{3}age+error$$

Equation (1) is used to estimate RII, when the link function g(Y) = log(Y) and SII when the link function g(Y) = Y. The error term has a binomial distribution. The coefficient *β*_*1*_ is the coefficient of interest and expresses RII when the link function is log and SII when the identity link is used. Y = 1 for exposure to the risk factor under study and Y = 0 is no exposure, *β*_*1*_^*……*^*β*_*4*_ correspond to the relevant regression coefficients, *ridit* is the ridit-score (replaces educational level), and *survey* represents the cross sectional survey (survey was coded 1 for HUNT 1, 2 for HUNT 2 and 3 for HUNT 3). The RII can be interpreted as the *rate ratio* and the SII can be interpreted as the *rate difference* at the bottom and the top of the educational hierarchy.

Trends in RII and SII over time were assessed by the inclusion of the two-way interaction term ridit-score by survey for each of the IHD risk factors. Gender differences in RII and SII at each survey were assessed by inclusion of the two-way interaction term ridit-score by gender for each survey. Furthermore, to assess if RII and SII changed differently over time in men and women, the three-way interaction term ridit-score by gender by survey was included in the model along with all two-way interactions together with the variables ofgender, survey and age. A positive, and significant, coefficient for the 3-way interaction term would indicate a larger increase in RII (or SII) in men compared to women. P-values ≤ 0.05 (two tailed) were considered to be significant. Statistical analyses were performed using STATA version 11.2 (StataCorp LP, College Station, Texas, USA). See Additional file 1 for STATA commands used.

### Ethics

The Norwegian Data Inspectorate, the Regional Committee for Ethics in Medical Research and the HUNT Research Centre approved the protocols for the HUNT surveys and for this study. Participating subjects in the HUNT Study provided written consent.

## Results

Educational level increased over time in both genders, and in HUNT 3 there were more women with a tertiary level education than men (Table 1). Still, the proportion of those with a primary level of education was highest among women in all three surveys.

**Table 1:** Unadjusted characteristics of participants by educational level and year of survey in The Nord-Trøndelag Health Study (HUNT) (percentages in brackets)

	HUNT 1 (1984–86)		HUNT 2 (1995–97)		HUNT 3 (2006–08)	
**Women**						
Age, year						
40-49	4982	(51.3)	6753	(55.2)	4538	(47.4)
50-59	4725	(48.7)	5481	(44.8)	5022	(52.6)
Educational level						
Primary	4397	(45.3)	2698	(22.1)	1744	(18.2)
Secondary	4357	(44.9)	7223	(59.0)	4814	(50.4)
Tertiary	953	(9.8)	2313	(18.9)	3002	(31.4)
Total	9707	(100)	12234	(100)	9560	(100)
**Men**						
Age, year						
40-49	4880	(51.0)	6238	(54.4)	3821	(45.4)
50-59	4676	(49.0)	5213	(45.6)	4592	(54.6)
Educational level						
Primary	3563	(37.3)	2168	(18.9)	1335	(15.9)
Secondary	4777	(50.0)	6991	(61.6)	5081	(60.4)
Tertiary	1216	(12.7)	2292	(20.0)	1997	(23.7)
Total	9556	(100)	11451	(100)	8413	(100)

### Smoking

In women, smoking prevalence increased for all education groups from HUNT 1 to HUNT 3, and mostly among those with a primary level education (Table 2). Among men there was a decline in all groups, especially among those with a tertiary education.

**Table 2:** Age-standardized prevalence, RII*and SII** of current smoking and diabetes among women and men aged 40–59 years between 1984 and 2008 in The Nord-Trøndelag Health Study (HUNT), by level of education

	HUNT I (1984–86)	HUNT II (1995–97)	HUNT III (2006–08)	P for trend
**Current smoking**				
**Women**				
Educational level				
Primary	37.6	48.6	46.2	
Secondary	30.1	37.9	33.9	
Tertiary	17.5	19.6	22.3	
RII (95% CI)	2.00 (1.77-2.24)	2.57 (2.35-2.80)	2.55 (2.28-2.81)	0.001
SII (95% CI)	22.54 (18.92-26.17)	36.13 (32.96-39.31)	30.09 (26.74-33.45)	<0.001
**Men**				
Educational level				
Primary	40.4	42.9	35.7	
Secondary	35.4	34.2	26.6	
Tertiary	25.7	20.1	17.4	
RII (95% CI)	1.57 (1.41-1.73)	2.31 (2.07-2.54)	2.48 (2.11-2.85)	0.000
SII (95% CI)	17.05 (13.26-20.84)	28.65 (25.30-32.00)	23.24 (19.59-26.89)	0.048
**Diabetes**				
**Women**				
Educational level				
Primary	1.2	1.3	2.2	
Secondary	1.4	1.5	2.5	
Tertiary	0.9	1.2	1.8	
RII (95% CI)	1.03 (0.32-1.74)	1.11 (0.47-1.75)	1.36 (0.70-2.00)	0.585
SII (95% CI)	−0.35 (−1.24-0.54)	0.01 (−0.89-0.90)	0.46 (−0.62-1.54)	0.053
**Men**				
Educational level				
Primary	1.4	2.4	4.1	
Secondary	1.5	2.1	3.3	
Tertiary	0.6	1.7	2.6	
RII (95% CI)	1.35 (0.48 -2.21)	1.38 (0.70-2.06)	2.00 (1.09-2.92)	0.201
SII (95% CI)	0.31 (−0.63-1.26)	0.50 (−0.40-1.38)	1.33 (0.01-2.66)	0.010

* Relative Index of Inequality.

**Slope Index of Inequality.

Educational inequalities in smoking increased both on the absolute scale (SII) and on the relative scale (RII) for both genders over the period covered by our three surveys. The overall absolute inequalities were larger among women compared to men in the second (p = 0.01) and the third (p = 0.01) surveys. Development in inequalities over time was similar for men and women both on the relative and absolute scale.

### Diabetes

The prevalence of diabetes increased significantly in all education groups, especially from HUNT 2 to HUNT 3 (Table 2). The overall test for trends showed stable relative inequalities in women (p = 0.59) and men (p = 0.20). Although the test for overall trend in relative inequalities in diabetes was not statistically significant in men, RII increased from 1.35 (0.48–2.21) in HUNT 1 to 2.00 (1.09–2.92) in HUNT 3. There were no gender differences in RII or SII in any of the three surveys. The test for overall trend in absolute inequalities was significant in women (p = 0.05) and men (p = 0.01), indicating widening absolute educational inequalities in diabetes in both genders over the last three decades. There were no gender differences in relative or absolute inequalities in any survey and the development of inequalities over time was similar for men and women both on the relative and absolute scale.

### Hypertension

The prevalence of hypertension declined substantially for all education groups from HUNT 1 to HUNT 3. In women, the largest reduction was observed among those with a primary level education, from 46% in HUNT 1 to 31% in HUNT 3 (Table 3). In men, the decline in prevalence of hypertension was greater among those with a secondary level education (from 54% in HUNT 1 to 37% in HUNT 3). In women, relative inequalities widened (p < 0.001), while absolute inequalities narrowed (p = 0.05). Also, in men relative inequalities widened (p = 0.01), while absolute inequalities were stable over the study period. Relative inequalities were significantly higher in women than in men in all surveys (test for gender difference: p < 0.001 for HUNT 1–3). There were larger absolute inequalities in women compared to men in HUNT 1 (p < 0.001) and in HUNT 2 (p < 0.001). Absolute inequalities increased more in men than in women over time (p-value for the three-way interaction term ridit-score by gender by survey was 0.02 for SII).

**Table 3:** Age-standardized prevalence, RII*and SII** of hypertension and high total cholesterol among women and men aged 40–59 years between 1984 and 2008 in The Nord-Trøndelag Health Study (HUNT), by level of education

	HUNT I (1984–86)	HUNT II (1995–97)	HUNT III (2006–08)	P for trend
**Hypertension**				
**Women**				
Educational level				
Primary	46.4	42.1	31.3	
Secondary	39.9	36.0	29.2	
Tertiary	31.1	26.9	20.4	
RII (95% CI)	1.47 (1.34-1.61)	1.56 (1.42-1.70)	1.63 (1.45-1.81)	0.001
SII (95% CI)	17.71 (13.97-21.45)	18.37 (15.22-21.51)	14.14 (11.08-17.20)	0.046
**Men**				
Educational level				
Primary	56.6	51.7	40.5	
Secondary	54.4	47.9	36.7	
Tertiary	46.8	42.8	32.8	
RII (95% CI)	1.16 (1.08-1.24)	1.21 (1.12-1.30)	1.28 (1.14-1.41)	0.011
SII (95% CI)	9.07 (5.20 -12.92)	10.54 (6.92-14.15)	9.99 (5.90-14.08)	0.327
**High total cholesterol**				
**Women**				
Educational level				
Primary		86.8	75.3	
Secondary		82.5	72.1	
Tertiary		75.7	69.6	
RII (95% CI)		1.10 (1.07-1.13)	1.07 (1.03-1.12)	0.369
SII (95% CI)		9.18 (6.84-11.50)	5.96 (2.85-9.07)	0.866
**Men**				
Educational level				
Primary		87.6	73.3	
Secondary		85.2	75.3	
Tertiary		82.2	71.8	
RII (95% CI)		1.07 (1.04-1.10)	1.04 (0.99-1.09)	0.209
SII (95% CI)		5.95 (3.39-8.52)	2.94 (−0.81-6.69)	0.112

* Relative Index of Inequality.

**Slope Index of Inequality.

### High total cholesterol

The prevalence of high total cholesterol declined substantially in all education groups from HUNT 2 to HUNT 3 (Table 3). Among those with primary level education the prevalence decreased by12% in women and 14% in men from HUNT 2 to HUNT 3. Relative and absolute inequalities were stable over time both in women and men. There were larger relative inequalities in women than in men in HUNT 2 (p < 0.001) and in HUNT 3 (p = 0.02), as well as larger absolute inequalities in total cholesterol in women in HUNT 2 (p < 0.001) and in HUNT 3 (p = 0.01) than in men. RII and SII changed similarly over time in women and men.

## Discussion

During the last three decades, in The Nord-Trøndelag county of Norway, we found educational inequalities in IHD risk factors with higher levels among those with primary education. Diabetes prevalence increased in all groups, while smoking prevalence increased in women and decreased in men. High total cholesterol and hypertension decreased in all education groups. There were stable absolute educational inequalities in high total cholesterol and hypertension in men. Absolute educational inequalities in hypertension decreased in women. Further, our results suggest widening absolute educational inequalities in smoking and diabetes in both genders.

### Smoking

Our results of increasing educational inequalities in smoking after the 1980s are consistent with findings from other studies [33-36]. However, while some studies confirm stable trends in absolute inequalities in smoking in both women and men [36], results from other studies correspond to our results, suggesting widening absolute [33,35,37] and relative [37] inequalities in smoking, especially in women [38]. The more unfavourable trend in smoking patterns in women [39] corresponds to comparative studies that confirm that educational inequalities in smoking are higher in women in northern Europe [16,40,41], particularly in Norway [40]. Large relative inequalities in ischaemic heart disease mortality in Norwegian women are evident [2,3] and smoking is a stronger risk factor for myocardial infarction in women compared to men [13] . Thus, a possible mechanism behind the steeper social gradient in ischaemic heart disease mortality is the less favourable trend in smoking patterns in women compared to men in Norway [40,42]. According to Lopez [43], the prevalence of smoking in developed countries can be referred to as a diffusion process. In the first stage, a new habit is most prevalent in higher socio-economic groups. In stage two the habit becomes more prevalent in all socio-economic groups. Rates among women also rise but lag behind those of men. In the third stage women reach their peak while prevalence rates start to decline among men, especially among higher socio-economic groups. In stage four prevalence rates keep declining, but at the same time socio-economic inequalities increase. Further, changes in smoking prevalence will affect smoking-attributable mortality three to four decades later [43]. Thus, it can be expected that social inequalities in mortality will persist for the next decades.

Still, due to societal changes, differences in health related behavior between women and men are narrowing, and results suggest that age and educational level are more important to a healthy lifestyle than gender [44]. Thus, preventing and reducing smoking among young people and the less educated should be a priority of policies aiming to reduce inequalities in IHD mortality.

### Diabetes

In accordance with findings of worldwide trends of increased prevalence of diabetes following a social gradient [10,35], levels of diabetes increased across all education levels in both genders in our study, and mostly among men with a primary level education. Our results correspond with a recent study of four Scottish Health Surveys between 1995–2008 [36]; however, our findings are only partly in line with Imkampe and Gulliford [45] who in a study of four cross sectional surveys in England between 1994 and 2006 found no association between educational level and diabetes in men, but increasing absolute and relative inequalities in women. Further, two recent cross sectional studies, one from the USA [35] and one from Spain [46] did not find any evidence for a widening trend in absolute inequalities in diabetes; nevertheless, in the study from the USA there was observed a considerable increase in diabetes prevalence across all education groups from 1971–2002. However, results from the two latter studies are not quite comparable with our study as analyses were not stratified by gender.

Diabetes prevalence increases with age, and the distribution of prevalence of diabetes will depend on the distribution of age across populations. The populations in our study were 40–59 years of age, and significantly narrower than in most other studies. Further, diagnosing practice and awareness of diabetes may differ across educational groups and between genders. However, in a recent systematic review on social inequalities in diabetes in countries with universal health care systems, Rici-Cabello et al. [47] did not find any support for gender differences in diagnoses and in the control of diabetes.

It is important to take into account that prevalence of risk factors for diabetes differs geographically and that this might produce different results between studies. Actually, recent results from the northern Sweden MONICA Study [48] suggest a stable trend in self-reported diabetes between 1986 and 2009. Nevertheless, in a cross-sectional study on social inequalities in diabetes across 13 European countries, Espelt et al. [10] found that educational inequalities in diabetes mortality were higher than inequalities in diabetes morbidity in the majority of countries included. The authors suggest that the mechanism behind this result is that factors related to disease progression (e.g., lower level of diabetes control and less access to and use of healthcare services) are more strongly related to social position than diabetes morbidity. As diabetes appears to be a strong predictor for IHD development [7], especially in women [8,9], our results underscore the need for public health efforts to turn the negative trend of an overall increase in diabetes prevalence and targeted interventions towards the least educated.

### Hypertension

Our results on decreasing prevalence of hypertension corresponds to results from a World Health Organisation project (WHO MONICA) on ten-year trends in IHD risk factors (1979–1996) in 21 countries from four continents [39]. Other studies also support decreasing levels of hypertension [34,35] across all education groups.

Relative inequalities in hypertension among men in our study were significantly widening; however, there are conflicting results about educational trends in hypertension. While some studies suggest stable trends in absolute inequalities in hypertension in both genders [33,36,49], Peltonen et al. [38] found stable absolute inequalities in hypertension among men and increasing absolute inequalities among women in the Northern Sweden MONICA Study from 1986 to 1994. Nevertheless, we have not been able to find any studies confirming our result of narrowing absolute inequalities in hypertension in women.

Despite an association between social position and hypertension [50], these inequalities seem to be less affected by social inequalities in treatment and control [33,51,52], which suggests that changes in blood pressure levels mainly arise from primary prevention such as a reduction in dietary intake of sodium [53] and saturated and trans fats [54]. Thus, cultural factors and difference in national public health strategies in primary prevention might contribute to the inconsistent findings in the social trends of hypertension in different countries. In a Norwegian longitudinal population-based study on educational inequalities, Strand et al. [55] found increasing absolute inequalities in systolic blood pressure among women from 1974–1988, which is in line with results from a recent American longitudinal study of Loucks et al. [56] from 1971–2001. These studies are not comparable to results from studies with a cross sectional design as in our study, however; the findings are important in relation to understanding lifetime trends and gender differences in the association between education and hypertension. Interestingly, in the study of Loucks et al.[56] educational level was inversely associated with blood pressure medication use in women but not in men. Further, the association between education and longitudinal trajectories of blood pressure did not diminish after adjustments for classical risk factors including antihypertensive use. Thus, the authors suggest [56] that psychosocial factors may be a mechanism behind the association between low education and hypertension.

Despite an overall decline in hypertension, recent results on global trends in systolic blood pressure show that women and men in western Europe have the highest systolic blood pressure in high income regions [57]. Thus, a future decrease in prevalence, as well as narrowing educational inequalities in hypertension prerequisites a frequent population-based monitoring of blood pressure levels and the use of hypertensive medication across all educational levels.

### High total cholesterol

Our results on the decreasing prevalence of high total cholesterol also correspond to results from the WHO MONICA study on ten-year trends in IHD risk factors (1979–1996) in 21 countries from four continents [39]. In addition, findings from other studies also support decreasing levels of total cholesterol [35,38,58,59] across all education groups. Further, our findings correspond to other studies suggesting stable absolute [33,49] and relative [49] inequalities in high total cholesterol among women and men. However, a Norwegian longitudinal study by Strand et al. [55] found that absolute educational inequalities in total cholesterol diminished in men during the study period (1974–1988) while they were stable among women. Despite the difference in study design, results from five cross-sectional studies from the northern Sweden MONICA study (1986–2004) [59] are in line with the longitudinal findings of Strand et al. [55], showing that the decline in total cholesterol was more rapid among men with low educational levels between 1990 and 1999 (narrowing educational inequalities). However, this trend reversed in a Swedish study [59] between 1999 and 2004, with an increase in cholesterol levels among men with primary and secondary educational levels at the same time as cholesterol levels continued to decrease among university-educated men (widening educational inequalities).

In Norway, the reduction of high total cholesterol levels is mostly attributed to dietary changes [54]. Nevertheless, according to Bartholomeeusen et al. [60], trends in cholesterol levels are also influenced by prescribed lipid-lowering drugs in general practice and changes in medical care, e.g., more patients are treated at lower cholesterol values now than in previous years. Thus, the observed trend in total cholesterol levels is probably affected by trends in medical practice. Further, prescriptions of statins may be one of the driving forces behind trends in educational inequalities in high total cholesterol. In fact, in a recent prospective population-based study of Norwegian women and men, Selmer et al. [61] found that in patients with no history of cardiovascular disease or diabetes, the start of statin treatment was not associated with educational level. However, in patients with a history of cardiovascular diasease or diabetes, those with a higher education, especially women, tended to start statin treatement more often than their counterparts with lower educational levels [61]. These findings are in accordance with those of Espelt et al. [10], suggesting that the mechanisms behind educational inequalites in disease ateiology may differ from the mechanism involved in social inequalities in prognoses as measured through cause-specific mortality.

Despite a marked decrease of cholesterol levels over the last decades, global trends show that serum total cholesterol levels are highest in high-income regions such as western Europe [62]. Recent results from a population-based study [63] also suggest that smoking potentiates the harmful effect of total cholesterol on risk of IHD, especially in women. Thus it is necessary to continue the assessment of cholesterol trends in realation to statin use, dieatary changes and smoking. Further, our results showing stable educational inequalites in high total cholesterol in women and men, calling for further public health initiatives to reduce educational inequalites in IHD development.

### Strengths and limitations of the study

The present study is based on three comparable and well designed surveys during three decades in a total population with high to acceptable response rates. Hypertension and total cholesterol were measured in a standardised manner. In addition, data on educational level in all three surveys was retrieved from a national high quality register in Statistics Norway.

In line with results from other population based health surveys, nonparticipation rates have been increasing over the last decades [64]. In our study, participation rates for those aged 40–59 decreased from 90% in HUNT 1 to 60% in HUNT 3. Results from a drop out study from HUNT 2 [65] revealed higher dropout rates for people with high alcohol consumption; abstainers and people with poor mental health while smoking was a predictor for non-participation across all analyses. In HUNT 3, dropout rates were higher for adults and elderly with somatic diseases and low social position [66]. Additionally, as life style factors and low social position are associated with nonparticipation in epidemiological studies [67], it seems possible that we have underestimated educational inequalities in IHD risk factors, especially in the last survey (HUNT 3). Further, as socioeconomic and health profiles differ in non-responders and responders, declining response rates over time will bias estimators of population trends [68]. In addition, there exist regional and national differences in IHD risk factor levels [69], and even similar educational gradients in IHD mortality between countries can be related to a non-uniform distribution of risk factors [70]. Our study population covers only one county in Norway. Nord-Trøndelag county is fairly representative of Norway regarding age distribution, economy, industry, morbidity and mortality [23]. However, the prevalence of higher education, and the prevalence of current smokers are a little lower than the average of Norway [23]. Thus, our findings may not necessarily be generalisable to the entire country.

Furthermore, the SII is sensitive to the average level of health in the population. If the prevalence rate increases in the same proportion in all the education categories, the SII will increase, whereas the relative differences remain constant [71]. Our assessment of smoking and diabetes depended on self report. Still, studies support that there is agreement between objective measurements and self reported diabetes [72] and smoking [73]. Lack of measurement of the use of lipid lowering drugs might have lead to an underestimation of levels of high total cholesterol; however, a Swedish study [58] did not find support for a relationship between increased use of lipid lowering medication and significantly reduced total cholesterol levels.

## Conclusions

During the last three decades in The Nord-Trøndelag county of Norway, we found educational inequalities in IHD risk factors with higher levels among those with primary education. Diabetes prevalence increased in all groups, while smoking prevalence increased in women and decreased in men. High total cholesterol and hypertension decreased in all education groups. There were stable absolute educational inequalities in high total cholesterol and hypertension in men while absolute educational inequalities in hypertension decreased in women. Further, our results suggest widening absolute educational inequalities in smoking and diabetes in both genders. The mechanisms behind these results are less clear, and future studies are needed to assess if educational inequalities in secondary prevention of IHD are larger compared to educational inequalities in primary prevention of IHD. The results emphasise the need for public health efforts to avoid further widening in socioeconomic inequalities in IHD mortality in Norway, especially among women.

## Competing interest

The authors declare that they have no competing interest.

## Author’s contributions

LE initiated the study, performed the statistical analysis, interpreted results and drafted the first version of the manuscript. BHS performed the statistical analysis, interpreted the results and helped to draft the manuscript. SMN contributed with critical revisions to the manuscript. GAE contributed with critical revisions to the manuscript. SK conceived the idea and contributed with critical revisions to the manuscript. All authors read and approved the final manuscript.

## Pre-publication history

The pre-publication history for this paper can be accessed here:

http://www.biomedcentral.com/1471-2458/12/266/prepub

## Supplementary Material

Additional file 1 STATA commands used in the paper to estimate Relative Index of Inequality (RII) and Slope Index of Inequality (SII). Click here for file
