# Supplementary material for: Trends in absolute and relative educational inequalities in four modifiable ischaemic heart disease risk factors: repeated cross-sectional surveys from the Nord-Trøndelag Health Study (HUNT) 1984–2008
Source: BMC Public Health. 2012 Apr 3;12:266. doi: 10.1186/1471-2458-12-266 (PMC3434116; doi:10.1186/1471-2458-12-266)
Supplement: Additional file 1 — STATA commands used in the paper to estimate Relative Index of Inequality (RII) and Slope Index of Inequality (SII). [file 1471-2458-12-266-S1.pdf]

Additional file 1

STATA commands used in the paper to estimate Relative Index of Inequality (RII) and Slope Index of Inequality (SII)

Data is on individual format (not aggregated), like this:

ID	Men	Survey	Smoking	Ridit-score	Age
1	1	1	0	0.34	53
2	0	1	1	0.55	48
3	0	2	0	0.34	55
4	1	3	1	0.34	40

Estimating Relative Index of Inequality (RII)

In the following example we estimated RII for smoking in men at the first survey, HUNT 1 (1984-86):

xi: glm smoking ridit age if survey==1 & men==1, fam(bin) link(log) nolog eform

where smoking=1 for smokers and 0 for non-smokers; ridit is the ridit score calculated as; age is age in years; survey=1 for HUNT1, 2 for HUNT2, etc; men=1 for men and 0 for women. The estimated coefficient for ridit gives the RII directly with 95% confidence interval.

Estimating Slope Index of Inequality (SII)

Similarly, SII for smoking was estimated:

xi: glm smoking ridit age if survey==1 & men==1, fam(bin) link(identity)

The only difference in coding for RII and SII is the link function, which is log for RII and Identity for SII. When link(identity) is used (as above) the estimated coefficient for ridit is the SII.

Estimating trend in RII and SII over time

Trend was estimated by the inclusion of the two-way interaction term ridit-score by survey (ridit_survey=ridit*survey)

- In the following example we estimated trend in RII for smoking in men:

xi: glm smoking ridit survey ridit_survey age if men==1, fam(bin) link(log) nolog eform

- and similarly SII for smoking in men:

xi:glm smoking ridit survey ridit_survey age if men==1, fam(bin) link(identity)

Estimating gender differences in RII and SII at each survey by inclusion of the two-way interaction term ridit-score by gender (ridit_men=ridit*men) in the model (analyses were stratified by survey):

xi:glm smoking ridit men ridit_men age if survey==1, fam(bin) link(log) nolog

- and similarly gender differences for SII :

xi:glm smoking ridit men ridit_men age if survey==1, fam(bin) link(identity)

Estimating if RII and SII changed differently over time in men and women, by inclusion of the three-way interaction term ridit-score by gender by survey (ridit_men_survey=ridit*men*survey) in the model:

xi:glm smoking ridit men survey ridit_men ridit_survey men_survey ridit_men_survey age, fam(bin) link (log) nolog

- and similarly for SII:

xi:glm smoking ridit men survey ridit_men ridit_survey men_survey ridit_men_survey age, fam(bin) link (identity) nolog
